# Notes and News

**Published:** 1896-04-04

**Authors:** 


### Notes and News.

(Collected and Communicated.)

HOSPITAL MEETINGS.

The Right Hon. Lord Kinnaird has consented to preside at a Festival Dinner in aid of the North London Hospital for Consumption to be held at the Hotel Cecil on Tuesday, June 9th.

The Duke of Westminster and Mr. C. G. Arbuthnot have each sent a contribution of £100 towards the building fund of the Grosvenor Hospital for Women and Children.

H.R.H. the Duke of York, who presided at the recent festival dinner of the Victoria Hospital for Children, Chelsea, has consented to become a vice-patron of the charity, of which H.R.H. the Duchess of York is also a vice-patron.

Since the exhibition of that most popular picture, "The Doctor," Mr. Luke Fildes has become quite the artist of the medical profession. In the forthcoming exhibition of the Royal Academy, his portraits of Dr. Buszard and Mr. Treves will appear.

Besides the memorial to be erected by inhabitants of the Isle of Wight collectively to the memory of the late Prince Henry of Battenberg, Ventnor proposes to establish one also. This will probably take the form of extended accommodation at the National Hospital for Consumption.

On Friday in last week an interesting ceremony took place at the Liverpool Stanley Hospital, when the Earl of Derby unveiled a stained-glass memorial window presented to the hospital by the tenantry on the Knowsley estate in memory of the fourteenth Earl of Derby, who first granted land to the original founders of the hospital, and allowed it to bear the family name of Stanley. The window, which was universally admired, is a most artistic and finished production, and lends a very pleasing effect to the principal entrance of the hospital.

The Princess of Wales has signified to the authorities of the West London Hospital, Hammersmith Road, her intention to open the bazaar and fête in aid of the building fund of that charity, at a date to be hereafter determined by her.

Many alterations and improvements are in progress at the Leeds General Infirmary. A clinical lecture theatre has already been provided, whilst necessary alterations and additions to the operation theatres are in progress. It is hoped by the end of the year all will be completed. It is also intended to erect a much-needed home for the nurses.

It is not very generally known that there are a certain number of lepers in Canada. At Tracadie a new lazaretto is being built, and to this it is proposed to transfer the patients now in British Columbia. There will then be thirty-four lepers in residence at Tracadie. The doctors who have been in charge of the hospital there render testimony to the temporary benefit of a treatment by Faith. They administered coloured water in place of drugs, and in nearly all cases a marked improvement was visible for a time, but the progress of the disease appeared to be all the more rapid afterwards.

A Buisson Institute for the prevention and cure of hydrophobia by vapour bath has been established in Norwood. In the meantime, we learn that thousands of stray dogs have been destroyed since the muzzling order has been established in the metropolis, and that not one was suffering from rabies. In another part of the country where the order is not in force hunting has been suspended, owing to an outbreak amongst a pack of hounds. There appears to be very little gained by regulations which allow the muzzled dog in one county to be bitten by an unmuzzled and rabid dog in the next, thus endangering the lives of, at any rate, the owners of the muzzled dog, roaming at large and unsuspected in the house. Legislation, which so inadequately protects the public, is an annoyance rather than a benefit.

We are glad to see that the *English Magazine* and the *Sketch* are both bringing before the public the facts relating to dogs and other animals trained for public performance. People like to believe that kindness is resorted to as the most efficacious way to produce the astonishing and amusing feats by animals which they witness at places of amusement. Careful inquiry, however, proves this not so, and clearly shows that the animals are subjected to frightful cruelty. The majority are trained abroad and bought in troupes by exhibitors, who maintain the same barbarous system of discipline. The proprietors of the English stage are not accused of connivance in any way, and, indeed, hardly any permit cruelty actually on the stage, but they have no control at rehearsals, and then it is that the poor animals are made to suffer. If the English public are as humane as they are represented to be, they would never witness another performance where animals are concerned until a proper system of inspection is instituted. For our part we should like these performances abolished entirely.

